# The Major Chromoblastomycosis Etiologic Agent *Fonsecaea pedrosoi* Activates the NLRP3 Inflammasome

**DOI:** 10.3389/fimmu.2017.01572

**Published:** 2017-11-20

**Authors:** Raffael Júnio Araújo de Castro, Isaque Medeiros Siqueira, Márcio Sousa Jerônimo, Angelina Maria Moreschi Basso, Paulo Henrique de Holanda Veloso Junior, Kelly Grace Magalhães, Luiza Chaves Leonhardt, Stephan Alberto Machado de Oliveira, Pedro Henrique Bürgel, Aldo Henrique Tavares, Anamélia Lorenzetti Bocca

**Affiliations:** ^1^Laboratory of Applied Immunology, Department of Cellular Biology, Institute of Biological Sciences, University of Brasília, Brasília, Brazil; ^2^Laboratory of Immunology and Inflammation, Department of Cellular Biology, Institute of Biological Sciences, University of Brasília, Brasília, Brazil

**Keywords:** NLRP3 inflammasome, *Fonsecaea pedrosoi*, chromoblastomycosis, hyphae, macrophages, dendritic cells

## Abstract

*Fonsecaea pedrosoi* is the main etiologic agent of chromoblastomycosis (CBM), one of the most prevalent subcutaneous mycosis in tropical and subtropical countries. CBM is a poorly characterized chronic infection that commonly starts after transcutaneous inoculation of conidia and saprophytic hyphae of *F. pedrosoi*. Recently, we have shown that unlike conidia, hyphae and muriform cells (the parasitic morphotype) of *F. pedrosoi* promotes an intense inflammatory response pattern *in vivo*, which comprises the production of an inflammasome-derived cytokine, IL-1β. Nonetheless, the mechanisms underlying IL-1β production and maturation upon *F. pedrosoi* infection and its functional output in the course of CBM remains unknown. We show here that *F. pedrosoi* hyphae, differently from conidia, induce IL-1β secretion in both bone marrow-derived dendritic cells and macrophages. Using inhibitors and knockout cells, we demonstrated that the mechanisms underlying IL-1β production by hyphae-infected macrophages were dependent on dectin-1, -2, and -3 receptors and the Syk-NF-kB signaling pathway. Furthermore, *F. pedrosoi* promoted a NLRP3-dependent inflammasome activation, which required potassium efflux, reactive oxygen species production, phagolysosomal acidification, and cathepsin B release as triggers. IL-1β processing and release was mediated primarily by caspase-1 and, to a lesser extent, by caspase-8-dependent cleavage. Finally, we showed using a murine CBM model that *F. pedrosoi* elicits a NLRP3-regulated IL-1β and interleukin-18 release *in vivo*, but without NLRP3 inflammasome activation interfering in the course of the experimental infection.

## Introduction

Chromoblastomycosis (CBM) is a chronic, granulomatous, suppurative, and often debilitating cutaneous and subcutaneous mycosis, caused by dimorphic filamentous fungi belonging to the *Dematiaceous* family (1–3). Multiple dematiaceous fungi are related to the disease etiology; of these, *Fonsecaea pedrosoi* and *Cladophialophora carrionii* are the most frequently identified fungal species in human CBM skin lesions. This disease occurs worldwide; however, it is mostly prevalent in tropical and subtropical areas (4, 5). Clinically, CBM is characterized by the slow development of polymorphic skin lesions, such as nodules, warts, tumors, plaques, and scars, after inoculation of fungal propagules consisting of conidia and hyphal fragments into host skin, more frequently into lower limbs (6–8). During infection of mammalian host, these primarily saprophyte fungal forms undergo transformation into the intensely melanized and thick-walled muriform (sclerotic) cells, the parasitic morphotype of *F. pedrosoi* (4).

Although little is known about the immune response of the host to infection by *F. pedrosoi*, it has been credited that an adaptive response mediated by T helper (Th) cell types 1 and 17 might be protective against *F. pedrosoi* infection (9–11). In this scenario, the abrogation of IL-12p35 transcription in human dendritic cells, leading to Th1-deficient development by several *Fonsecaea* species, and the Th17-mediated response suppression in experimentally infected mice, suggest that this fungal pathogen evade host immune response by complex mechanisms. These mechanisms usually encompass the evasion or subversion of the function of innate pattern recognition receptors (PRRs) in the detection of conserved fungal components or pathogen-associated molecular patterns (PAMPs) by phagocytes (10–12). A number of PRRs families have been associated with *F. pedrosoi* sensing, including the C-type lectin receptors (CLRs) mincle, dectin-1 and dectin-2, as well as toll-like receptors (TLRs). Besides these cytoplasmic membrane-bound receptors, fungal sensing by cytosolic PRRs, such as NOD-like receptors (NLRs) and AIM2-like receptors, is becoming increasingly apparent.

The members of the NLR protein family typically share three functional domains: a C-terminal leucine-rich-repeat putative ligand-binding domain, a central NACHT nucleotide-binding and oligomerization domain and an N-terminal signaling domain (13). The latter consists of different domains, most notably a pyrin domain (PYD) or a caspase recruitment domain (CARD). Certain NLRP (NLR subfamily with an N-terminal PYD), such as NLRP1 and NLRP3, and the NLR family CARD domain-containing protein 4 (NLRC4) associate with inflammatory caspase-1 (in the form of pro-caspase-1) to assemble the inflammasome, a large cytosolic multiprotein complex. Notably, NLRP3-containing inflammasome formation is dependent on the adaptor protein ASC (apoptosis-associated speck-like protein containing a CARD), which promotes the recruitment of pro-caspase-1 through CARD–CARD interactions (13). The assembly of the inflammasome complex leads to the cleavage of pro-caspase-1 into an active cysteine protease, which cleaves the proinflammatory cytokines interleukin-1β (IL-1β) and interleukin-18 (IL-18) into their mature forms. NLRP3 inflammasome, the most studied and the main inflammasome associated with fungal infection, is activated by a typical two-step mechanism: priming and activation (14, 15). The priming signal is generated by the recognition of PAMPs by PRRs, usually leading to NF-kB activation and, as a result, production of pro-IL-1β, pro-IL-18, and NLRP3. The activation step is associated with the assembly of the multiprotein complex induced by a broad variety of endogenous danger-associated molecules (DAMPs), such as potassium efflux, production of reactive oxygen species (ROS), phagolysosome acidification, and cathepsin B release. Several conditions may lead to DAMPs production, including metabolic disorders, inflammatory diseases, and infections.

The inflammasome-dependent release of IL-1β and IL-18 cytokines has a striking importance in the regulation of innate and adaptive response against many different fungal pathogens, including significant protective roles against *Candida albicans, Aspergillus fumigatus, Cryptococcus neoformans, Paracoccidioides brasiliensis*, and *Histoplasma capsulatum* (16–22). One aspect associated with inflammasome activation is fungal morphotype diversity and complexity. *C. albicans* hyphae are better inducers and the only fungal form of *A. fumigatus* that activates the NLRP3 inflammasome (16, 17, 23). Indeed, we have recently showed that unlike conidia, the hyphae and muriform (sclerotic) cells of *F. pedrosoi* promote intense production of proinflammatory cytokines *in vitro* and *in vivo* (24). Among these cytokines, we observed IL-1β production, suggesting that *F. pedrosoi* could activate the inflammasome. In this context, we aimed to evaluate the inflammasome activation by *F. pedrosoi* infective propagules and the role of the inflammasome in CBM experimental disease.

We show here that *F. pedrosoi* hyphae, differently from conidia, induce IL-1β secretion in both bone marrow-derived dendritic cells and macrophages. The mechanisms underlying IL-1β production by macrophage-infected hyphae were dependent on dectin-1, -2, and -3 receptors and the Syk-NF-kB signaling pathway. *F. pedrosoi* promoted a NLRP3-dependent inflammasome activation, which required K^+^ efflux, ROS production, phagolysosomal acidification, and cathepsin B release. IL-1β processing and release were mediated by caspase-1 and, to a lesser extent, caspase-8-dependent cleavage. Furthermore, we demonstrated using an experimental CBM model that *F. pedrosoi* elicits a NLRP3-regulated IL-1β and IL-18 release *in vivo*. However, we did not observe an influence of the NLRP3 inflammasome on the control of the fungal infection.

## Materials and Methods

### Fungal Culture and Preparation

*Fonsecaea pedrosoi* ATCC 46428 were maintained in Sabouraud dextrose agar medium (SDA) at 37°C after serial animal passages to enhance fungal virulence. In order to obtain purified conidia and hyphae for experiments, virulent *F. pedrosoi* propagules were grown in potato dextrose medium in a rotary shaker (120 rpm) at 30°C. A 15-day-old suspension containing conidia and hyphal fragments was submitted to successive filtrations on 70 and 40 µm cell strainers (BD), respectively. The 40 µm filter-retained cells (ranging between 40 and 70 µm) were re-suspended in phosphate-buffered saline (PBS) and consisted of more than 98% of purified hyphae. The suspension of cells smaller than 40 µm was further filtered using a 14 µm filter paper (J. Prolab) to remove small hyphal fragments and achieve a minimum of 98% purified conidia. A mix of purified hyphae and conidia at a 3:1 rate was used as *F. pedrosoi* fungal propagules for *in vivo* assays. For assays with inactivated fungi, conidia, and hyphae were heat-killed by boiling for 40 min, or fixed with 3% paraformaldehyde (PFA) for 6 h. *F. pedrosoi* cells were washed twice, counted using a hemocytometer, and used for experiments.

### Cell Culture

Bone marrow-derived macrophages (BMDMs) and dendritic cells (BMDCs) were generated by a previously described method (25). Briefly, bone marrow cells were flushed out of murine femurs and tibias and submitted to erythrocyte lysis using tris-buffered ammonium chloride. Cells (2 × 10^6^) were plated onto non-tissue culture-treated Petri dishes in 10 mL of RPMI-1640 medium (Sigma-Aldrich) supplemented with 10% heat-inactivated FBS (Gibco), 50 µM 2-mercaptoethanol, 50 µg/mL of gentamicin, and 20 ng/mL GM-CSF (Peprotech), and cultured for 8 days in a humidified 5% CO_2_ atmosphere at 37°C. On day 3, another 10 mL of fresh complete medium was added to the culture. On day 6, half of the medium was exchanged. On day 8, loosely adherent/suspended BMDCs and firmly adherent BMDMs stripped with TrypLE™ Express (Gibco) were separately collected and plated at a density of 10^6^ cells/mL in RPMI medium containing 10% FBS and 50 µg/mL of gentamicin, for experimental use. THP-1 cells were maintained and used under the same experimental use conditions.

### *In Vitro F. pedrosoi* Challenge and Cell Treatments

Bone marrow-derived macrophages derived from wild-type (WT) or knockout mice were infected with *F. pedrosoi* for 6 (RT-qPCR analysis) and 24 h (other assays) at a multiplicity of infection (MOI) of 3 for conidial infection (except for fungicidal assays, performed with a MOI of 1) and of 1 for hyphal. For inhibition assays, BMDMs received 2 h prior to the infection a Myd88 inhibitor peptide (50 µM) (InvivoGen), Syk inhibitor R406 (5 µM) (InvivoGen), NF-kB inhibitor celastrol (5 µM) (InvivoGen), caspase-1 inhibitor AC-YVAD-CHO (50 µM) (Santa Cruz Biotechnology), caspase-8 inhibitor Z-IETD-FMK (50 µM) (Santa Cruz Biotechnology), intracellular potassium efflux inhibitors glyburide (150 µM) (InvivoGen) and KCl (50 mM) (Sigma-Aldrich), ROS inhibitor DPI (diphenyleneiodonium chloride) (20 µM) (Sigma-Aldrich), endosomal acidification inhibitor bafilomycin A (250 nM) (InvivoGen) or cathepsin B inhibitor CA-074 Me (50 µM) (Sigma-Aldrich). In some experiments, BMDMs and BMDCs were treated 2 h previously to the fungal infection with 500 ng/mL of LPS (Sigma-Aldrich) and/or 20 µM of nigericin (InvivoGen) during the last 40 min of incubation (1 h for fungicidal assays). Cells stimulated with both LPS and nigericin served as a positive control for NLRP3-mediated inflammasome activation.

### Analysis of Fungal Cell Morphology by Flow Cytometry

In order to induce and evaluate conidial swelling, *F. pedrosoi* conidia (5 × 10^6^ cells/mL) were incubated for 6 h with PBS or RPMI supplemented with 20% of heat-inactivated FBS at 37°C, under 120 rpm. In addition, conidia were incubated with BMDMs at a MOI of 3, for 6 and 24 h. Infected BMDMs were washed to discard nonphagocyted fungus and lysed with 0.05% SDS to release intracellular fungi. Then, fungal were fixed with PFA treatment, washed twice, and evaluated by flow cytometry analysis. Micron-size beads of 2 and 3 µm (CS&T Research Beads, BD) were used as control. Cell acquisition was performed using a FACSVerse (BD) flow cytometry, and data were analyzed with FlowJo v.10 software.

### *In Vitro* Fungicidal Assay

Bone marrow-derived macrophages derived from WT, *Nlrp3*^−/−^, and *Caspase-1/11*^−/−^ mice were infected for 24 h with *F. pedrosoi* conidia or hyphae. In addition, WT macrophages were also treated, or not, with nigericin as described above, or stimulated 3 h before the infection with LPS and IFN-γ (both from Sigma-Aldrich) (500 and 20 ng/mL, respectively) to activate the mechanisms of macrophage killing [e.g., nitric oxide (NO) production]. After infection, the cell culture supernatant was harvested for NO determination. The remaining cell monolayers were carefully washed to remove non-adherent cells, lysed as described above, and re-suspended in PBS. After serial dilutions, cell suspension was plated on SDA and incubated at 30°C for 5–7 days for colony-forming unit (CFU) evaluation.

### Detection of Activated Caspase-1 by Fluorochrome-Labeled Inhibitor of Caspases (FLICA)

After 24 h of infection with *F. pedrosoi* conidia or hyphae, BMDMs were detached (as mentioned above) and incubated for 1 h with a caspase-1 fluorochrome-labeled inhibitor of caspases (FLICA), FAM-YVAD-FMK (Immunochemistry Technologies), according to the manufacturer’s instructions. Next, cells were washed and stained with APC-conjugated anti-CD11b antibody (eBioscience) in PBS with 2% heat-inactivated FBS (Gibco) to distinguish macrophages from non-internalized fungus. Then, cells were washed and samples were analyzed by flow cytometry as described above.

### Quantitative Real-time PCR (qRT-PCR)

Total RNA from BMDMs was extracted using the TRIzol reagent (Invitrogen) and cDNA was synthesized using the high capacity RNA-to-cDNA kit (Applied Biosystems), according to manufacturer’s protocols. qRT-PCR was performed using SYBR green incorporation (Applied Biosystems) and real-time PCR equipment (StepOne system) (Applied Biosystems). Expression of the genes of interest was normalized to the expression of the housekeeping gene *Rps9* and expressed as “Fold change,” which was calculated by the 2^−ΔΔCT^ method (26). The primers used were validated according Livak and Schmittgen (26) and listed in Table S1 in Supplementary Material.

### Animals and *In Vivo F. pedrosoi* Infection

*Clec7a*^−/−^ (*Dectin-1*^−/−^) mice were supplied by Dr. Gordon Brown (University of Aberdeen, Scotland). *Clec4n*^−/−^ (*Dectin-2*^−/−^), *Clec4d*^+/+^ (*Dectin-3*^+/+^), *Clec4d*^−/−^ (*Dectin-3*^−/−^) mice were kindly provided by Dr. Bruce Klein (University of Wisconsin-Madison, USA). All animals (C57BL/6 background), including *Nlrp3*^−/−^, *Caspase-1/11*^−/−^, and C57BL/6 WT mice, were maintained under pathogen-free conditions and used at 8 to 12 weeks old for experiments. For *in vivo* infection, WT, *Nlrp3*^−^*^/^*^−^ and *Caspase-1/11*^−/−^ mice were inoculated subcutaneously into the hind footpad with 50 µl (per foot) of a suspension containing 1 × 10^6^ (2 × 10^7^/mL) *F. pedrosoi* hyphae and conidia in the proportion of 3:1, respectively (fungal propagules). Mice were euthanized at 14, 21, and 28 days postinfection and the footpad was collected, weighed, and homogenized for ELISA assay and plated for CFU analysis.

### Measurement of NO and Cytokine Production

Nitrite $\left({\text{NO}}_{2}^{-}\right)$ concentration in culture supernatants was applied as an indicator of NO generation and measured with the Griess reagent (1% sulfanilamide, 0.1% naphthylethylene diamine dihydrochloride, 2.5% H_3_PO_4_). For that, 50 µl of the culture supernatant was added to an equal volume (v/v) of Griess reagent and allowed to incubate at room temperature for 10 min. Absorbance was measured at 540 nm using a microplate reader. The ${\text{NO}}_{2}^{-}$ concentration was determined using a standard curve of 1.56–100 µM of NaNO_2_. Cytokine levels from the homogenized animal tissue and cell culture lysate and supernatants were determined by ELISA, according to the manufacturer’s guidelines, for the following cytokines: human interleukin-1β (IL-1β) and murine IL-1β (both uncleaved and cleaved forms), IL-18 and tumor necrosis factor-α (TNF-α), all purchased from eBioscience. Results were expressed as cytokines pictogram per milliliters or per 100 mg of tissue, for samples obtained from *in vivo* assays.

### Statistical Analysis

Statistical analysis was conducted using GraphPad Prism v.5.0 software. Data were analyzed by one-way ANOVA followed by Tukey’s *post hoc* test. Two-way ANOVA and Bonferroni’s *post hoc* test were used to compare different groups with more than one variable. *p-*Values of less than 0.05 were considered significant.

## Results

### *F. pedrosoi* Hyphae, but Not Conidia, Induce IL-1β Secretion in BMDMs and THP-1 Cells

**Figure 1:**
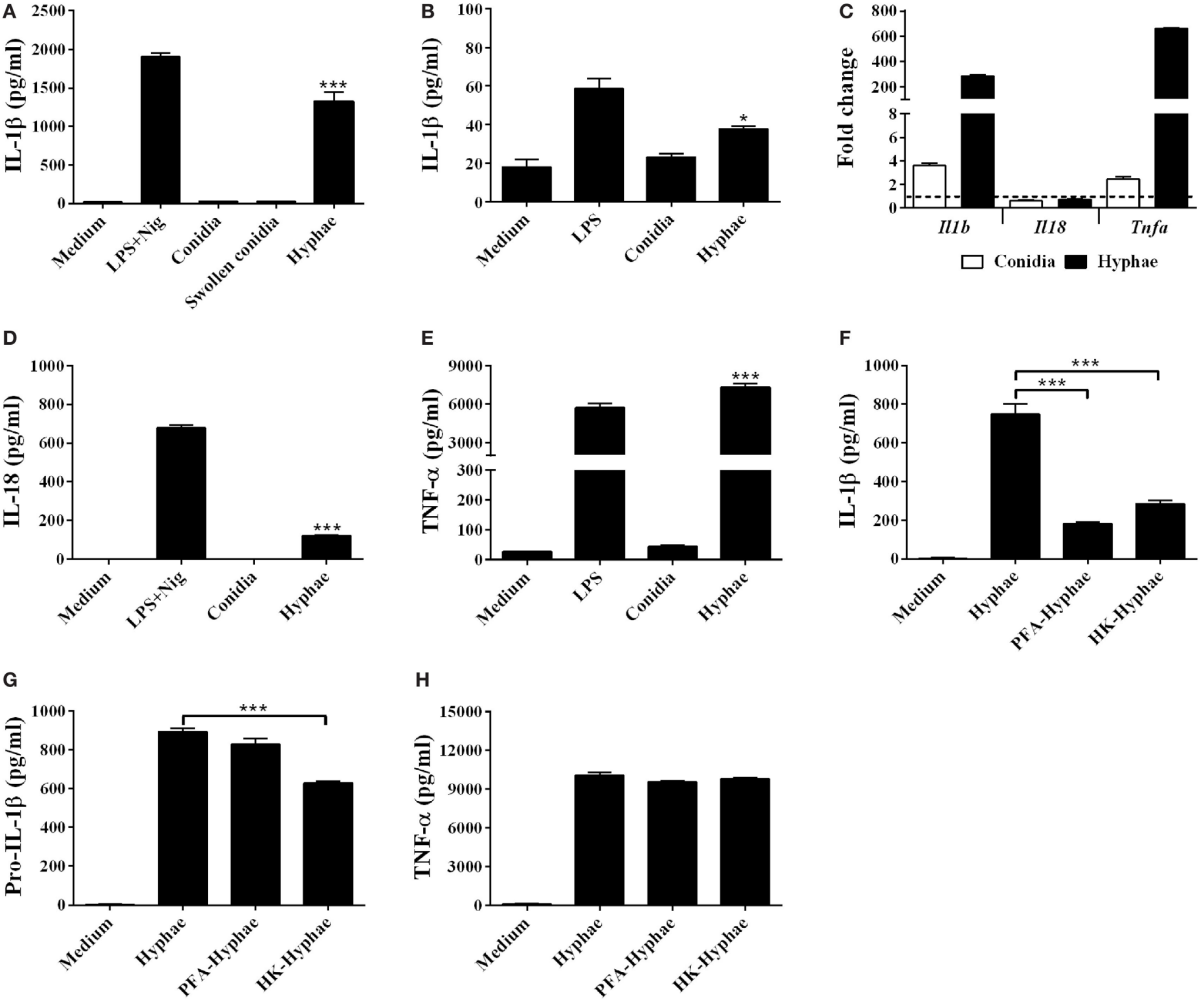
*Fonsecaea pedrosoi* hyphae, but not conidia, induce IL-1β secretion in bone marrow-derived macrophages (BMDMs) and THP-1 cells. IL-1β levels after 24 h of BMDMs incubation with medium, *F. pedrosoi* conidia, swollen conidia, or hyphae **(A)**. IL-1β secretion after 24 h of THP-1 cell incubation with medium, *F. pedrosoi* conidia, or hyphae **(B)**. Intracellular *Il1b, Il18*, and *Tnfa* gene transcription of BMDMs after 6 h stimulation with hyphae or conidia were analyzed by RT-qPCR **(C)**. Interleukin-18 (IL-18) **(D)** and tumor necrosis factor-α (TNF-α) **(E)** secretion of BMDMs co-cultivated with medium, conidia, or hyphae. BMDMs were stimulated also with live, paraformaldehyde-killed, or heat-killed (HK) hyphae for IL-β **(F)**, pro-IL-β **(G)**, and TNF-α measurement **(H)**. Pro-IL-1β was evaluated in the cell lysate after 12 h of infection, while the others cytokines were assayed in the cell supernatant after 24 h. IL-1β, pro-IL-1β, IL-18, and TNF-α levels were evaluated by ELISA. Data shown are mean ± SEM and are representative of two to three independent experiments. *p < 0.05; ***p < 0.001, compared to cells with only medium or between groups indicated by brackets.

To determine whether *F. pedrosoi* could induce IL-1β secretion, we infected murine BMDMs with *F. pedrosoi* conidia or hyphae for 24 h. Hyphae cell infection resulted in IL-1β secretion, while conidia failed to induce significant levels of this cytokine (Figure 1A). In addition, THP-1, a human monocyte cell line, secreted IL-1β upon infection by *F. pedrosoi* hyphae, but not conidia (Figure 1B). To test if the size of conidia could affect the BMDM activation, we induced swollen-conidia (Figure S1 in Supplementary Material), an intermediate stage of conidia-into-hyphae transformation, and used it to stimulate BMDMs. Swollen conidia were equally unable to promote IL-1β secretion (Figure 1A).

Then, we evaluated if the lack of secreted IL-1β in BMDM-conidia coculture supernatant results primarily from the inability of BMDMs to trigger *Il1b* gene transcription after conidial infection. RT-qPCR analysis revealed that at 6 h of infection, hyphae and, to a lesser extent, conidia induce *Il1b* and *Tnfa*, but not *il18* transcripts in BMDMs (Figure 1C). ELISA assay performed with 24 h culture supernatant showed that, contrary to conidial, hyphal infection results in IL-18 and TNF-α secretion (Figures 1D,E), despite the observation that the *Il18* transcript is not upregulated. Further, we stimulated BMDMs with inactivated hyphae to determine whether the viability of this fungal form influences IL-1β secretion. Both PFA and heat-killed (HK) hyphae stimulus resulted in severe reduction of IL-1β in comparison to live fungus (Figure 1F), whereas the viability of the fungus did not significantly affect the levels of TNF-α production and only slighted affected the levels of pro-IL-1β only in the HK hyphae (Figures 1G,H). These finding suggest that that the viability of *F. pedrosoi* affects activation, but not priming of NLRP3 inflammasome.

### Differential Inflammasome Activation Requirements in BMDMs and BMDCs Are Morphotype-Dependent in *F. pedrosoi* Infection

**Figure 2:**
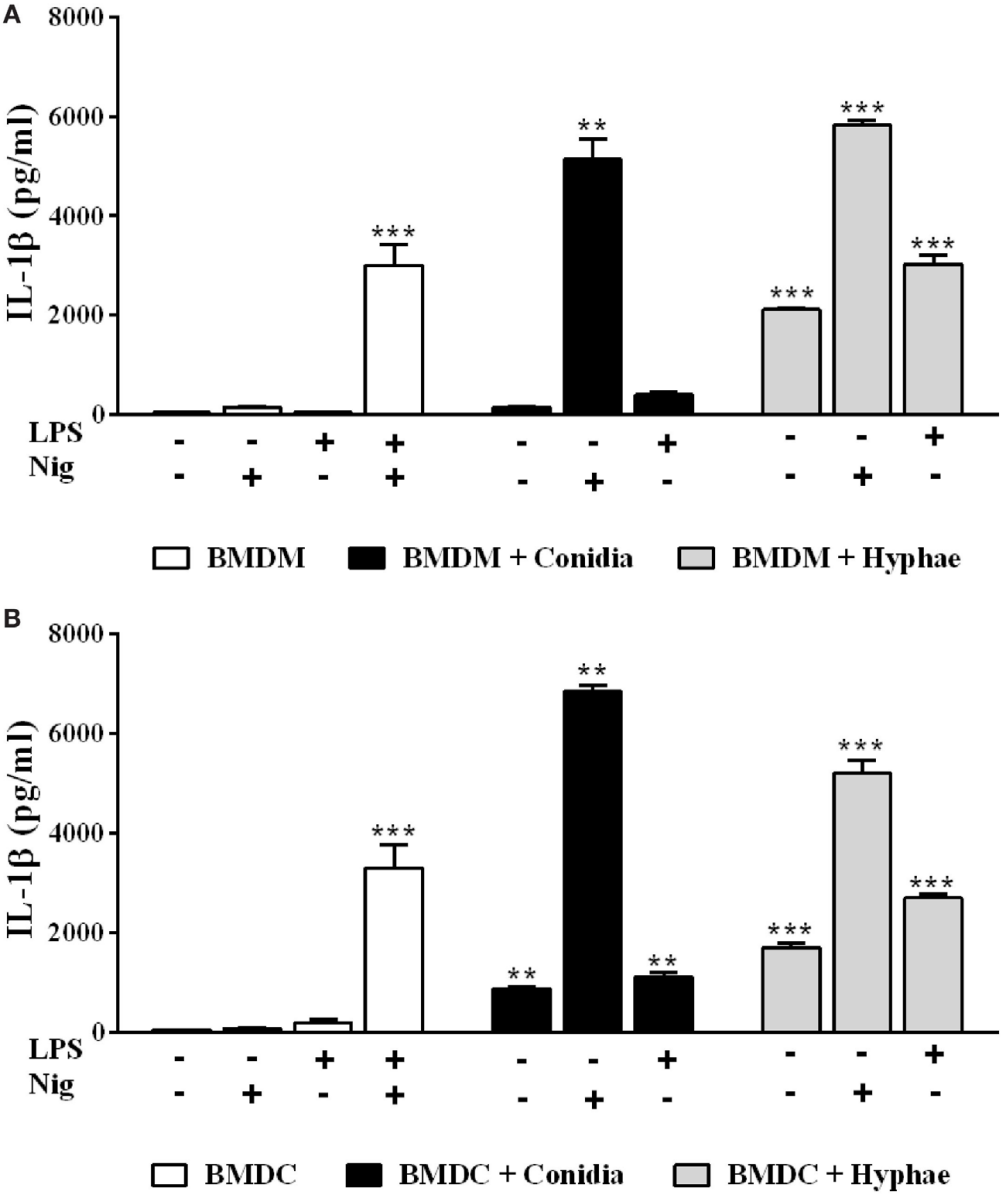
Differential inflammasome activation requirements in bone marrow-derived macrophages (BMDMs) and dendritic cells (BMDCs) are morphotype-dependent in *Fonsecaea pedrosoi* infection. IL-1β detection by ELISA in supernatants of BMDMs **(A)** and BMDCs **(B)** incubated for 24 h with medium, *F. pedrosoi* conidia or hyphae, and co-stimulated (or not) with LPS (500 ng/mL added 2 h before fungal infection), nigericin (20 µM during the last 40 min of incubation), or both (as a positive control). Data shown are mean ± SEM and are representative of three independent experiments. **p < 0.01; ***p < 0.001, compared to cells with only medium.

Since we previously showed that BMDCs differ from BMDMs regarding inflammasome activation requirements upon *P. brasiliensis* infection (27), we investigated whether this is also the case with *F. pedrosoi*. Differently from BMDM infection, conidia alone induced IL-1β secretion in BMDCs, suggesting that it acts as both the first and the second signal for inflammasome activation in this cell type (Figures 2A,B). When nigericin was added to BMDMs or BMDCs, these cells secreted a large amount of mature IL-1β, confirming that conidia elicit pro-IL-1β production (as indicated in Figure 1C), but fail to provide further inflammasome activating signals in BMDMs (Figures 2A,B). Regarding hyphae, this morphotype alone was able to induce IL-1β secretion not only in BMDMs (as shown in Figure 1) but also in BMDCs (Figure 2B).

### The Syk-NF-kB Signaling Pathway, Coupled to Dectin-1, Dectin-2, and Dectin-3 Receptors, Enables Inflammasome Activation by BMDMs Challenged with *F. pedrosoi* Hyphae

**Figure 3:**
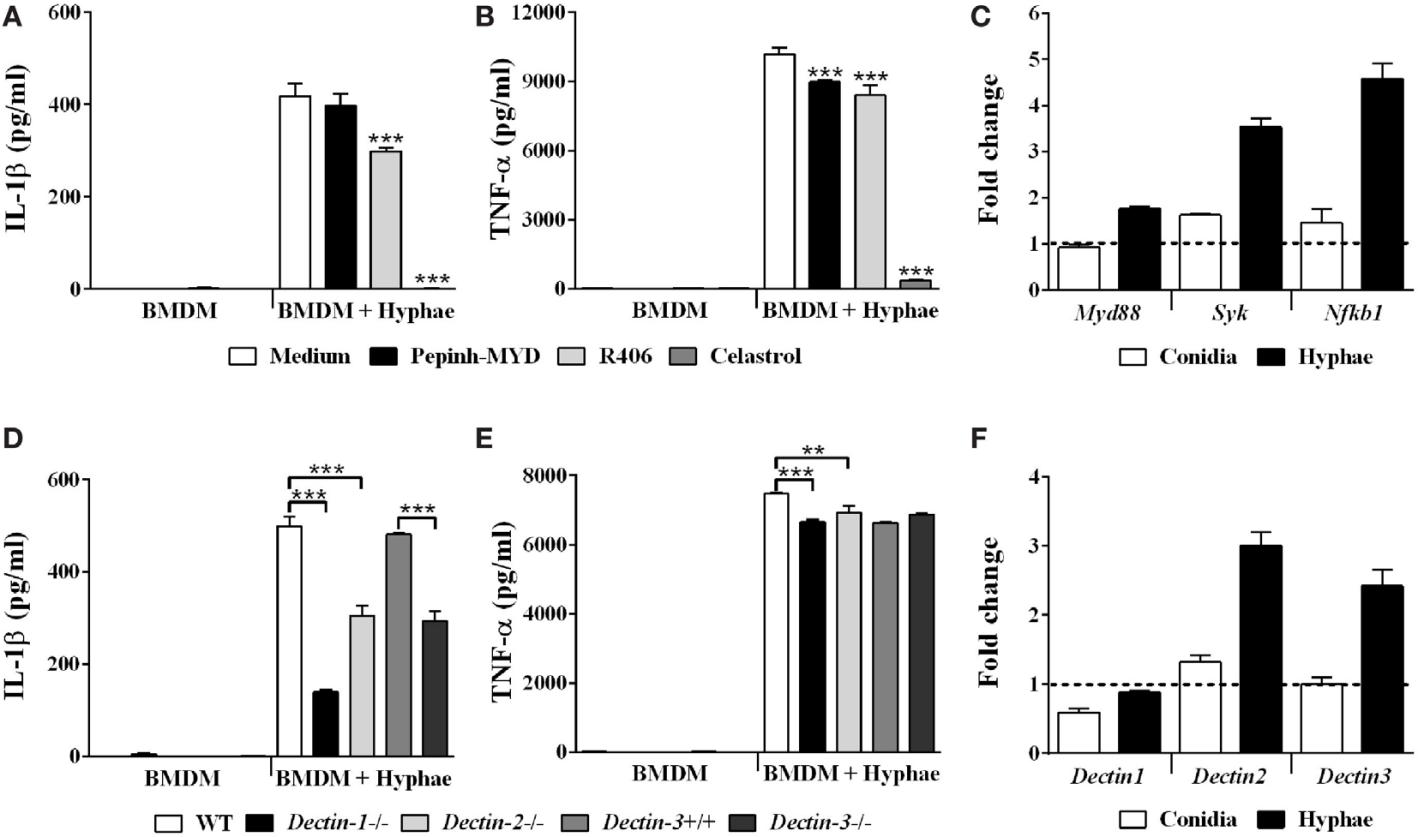
The Syk-NF-kB signaling pathway, coupled to dectin-1, dectin-2, and dectin-3 receptors, enables inflammasome activation by bone marrow-derived macrophages (BMDMs) challenged with *Fonsecaea pedrosoi* hyphae. BMDMs were pretreated for 2 h with Myd88 inhibitor peptide (50 µM), R406 (5 µM), and celastrol (5 µM) and thereafter infected with *F. pedrosoi* hyphae for 24 h **(A,B)**. BMDMs were infected with conidia or hyphae for 6 h, and then were lysed for RT-qPCR analysis to quantify gene expression of indicated genes **(C,F)**. BMDMs obtained from wild-type (WT), *Dectin-1^−/−^, Dectin-2^−/−^, Dectin-3^+/+^*, and *Dectin-3^−/−^* mice were infected with *F. pedrosoi* hyphae for 24 h **(D,E)**. The supernatant of 24 h cell culture assays were evaluated for IL-1β and TNF-α by ELISA. Data shown are mean ± SEM and are representative of two to three independent experiments. **p < 0.01; ***p < 0.001, compared to infected untreated cells or between groups indicated by brackets.

Macrophages play central roles in host immune response against fungal infections (28). Likewise, these phagocytes are not only ubiquitous in CBM lesions but are also actively involved in the recognition of fungal cells and in the modulation of inflammatory responses against *F. pedrosoi* (4, 29, 30). In this context, we performed subsequent assays focused on macrophage-hyphae interaction, since we show here that hyphae are the *F. pedrosoi* infective morphotype most likely to be responsible for initiating the inflammatory response observed in CBM. In order to determine the mechanisms underlying BMDMs inflammasome priming in *F. pedrosoi* infection, we treated BMDMs with celastrol, an inhibitor of IKK activity, which results in the inactivation of NF-kB, the main transcriptional factor associated with pro-IL-1β transcription. As expected, NF-kB inhibition led to a complete abolition of IL-1β, as well as a strong reduction of TNF-α secretion in cell supernatant (Figures 3A,B, respectively). Furthermore, it is known that NF-kB-activating PRRs license inflammasome activation by providing both pro-IL-1β and inflammasome receptors expression (31). To clarify the nature of the PRRs that are involved in the *F. pedrosoi* recognition that led to NF-kB-dependent BMDMs priming, we evaluated the transcription of *Myd88* and *Syk*, which are pivotal signal transducers of TLRs and CLRs, respectively. We observed that hyphae not only induced *Syk* and *Nfkb1* upregulation (Figure 3C), but also depend on Syk activity to induce IL-1β secretion, as shown in BMDMs treated with Syk inhibitor R406 (Figure 3A). *Myd88*, however, was poorly transcribed in BMDMs upon hyphal infection (Figure 3C), and its activity was indifferent to IL-1β secretion in cells treated with a Myd88 inhibitory peptide (Figure 3A).

It is known that CLRs, such as dectin-1, dectin-2, and dectin-3, signaling *via* Syk kinase promote NF-kB activation (32). To test whether these CLRs were responsible for recruiting Syk to signal inflammasome priming in BMDMs challenged with hyphae, we used cells from mice deficient in these receptors, and observed that *Dectin-1^−^*^/^*^−^, Dectin-2^−^*^/^*^−^*, and *Dectin-3^−^*^/^*^−^* BMDMs show reduced IL-1β secretion when compared to their respective controls (Figure 3D). Except for *Dectin-3^−^*^/^*^−^* cells, all tested knockout cells also exhibited a slight reduction in TNF-α levels (Figure 3E). Dectin-1 KO cells had the strongest depletion of IL-1β (Figure 3D), although WT BMDMs did not present differential regulation of *Dectin-1* transcription—only *Dectin-2* and *Dectin-3* genes were upregulated (Figure 3F). Further, since the inhibition of Syk signaling did not completely abrogate the IL-1B secretion (Figure 3A), we reasoned that an alternative Syk-independent dectin-1-NF-kB signaling pathway would be in use. To evaluate this hypothesis, we inhibited Raf-1 kinase signaling, an adaptor protein that dectin-1 engages to signal NF-kB activation independently of Syk (33); however, IL-1β levels were unaffected (data not shown). The results indicate that the Syk-NF-kB signaling pathway, coupled to dectin-1, dectin-2, and dectin-3 receptors, enables inflammasome activation by BMDMs challenged with *F. pedrosoi* hyphae.

### Inflammasome Activation by *F. pedrosoi* Hyphae Is Dependent on Caspase-1 and Caspase-8

**Figure 4:**
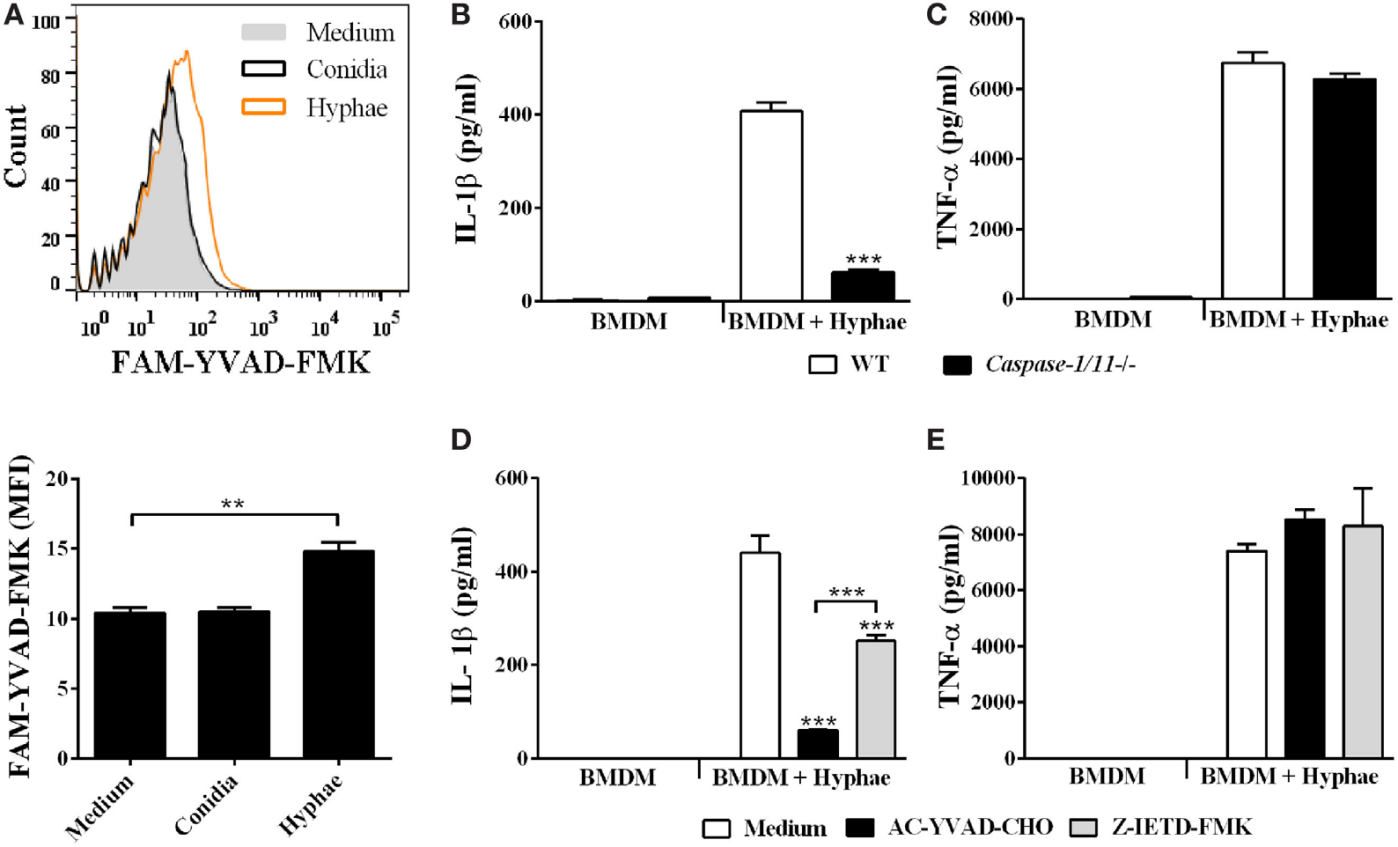
Inflammasome activation by *Fonsecaea pedrosoi* hyphae is dependent on caspase-1 and caspase-8. Bone marrow-derived macrophages (BMDMs) were incubated with medium, *F. pedrosoi* conidia or hyphae for 24 h, and thereafter stained with anti-CD11b-APC antibody and FAM-YVAD-FMK-FITC probe to perform flow cytometry analysis. Caspase-1 activity (indicated by intensity of FAM-YVAD-FMK-FITC binding) of CD11b^+^ cells is represented in histogram and by cell mean fluorescence intensity (MFI) **(A)**. BMDMs obtained from wild-type (WT) or *Caspase-1/11^−/−^* mice were infected with *F. pedrosoi* hyphae for 24 h **(B,C)**. WT BMDMs were pretreated for 2 h with 50 µM of AC-YVAD-CHO (caspase-1 inhibitor) or Z-IETD-FMK (caspase-8 inhibitor) and then infected with *F. pedrosoi* hyphae for 24 h **(D,E)**. The supernatant of cell culture assays **(B–E)** was evaluated for IL-1β and tumor necrosis factor-α by ELISA. Data shown are mean ± SEM and are representative of two to three independent experiments. **p < 0.01; ***p < 0.001, compared to WT-infected untreated cells or between groups indicated by brackets.

IL-1β secretion requires proteolytic cleavage of its inactive pro-form, canonically performed by caspase-1 enzyme. To assess whether *F. pedrosoi* infective morphotypes were able to activate caspase-1, we used a fluorescent probe, FAM-YVAD-FMK, which specifically binds the active protease, and analyzed stained BMDMs using flow cytometry. As expected, only hyphae, but not conidia, were able to induce caspase-1 activation (Figure 4A), which corroborates our ELISA data showing that only hyphae infection led to IL-1β processing and secretion in BMDMs (Figure 2A). Then, to confirm that IL-1β secretion was caspase-1 dependent, we challenged BMDMs from WT and *Caspase-1/11* knockout mice with hyphae. In addition, we also treated WT BMDMs with AC-YVAD-CHO, a specific caspase-1 inhibitor. Both *Caspase-1/11^−^*^/^*^−^* BMDMs and WT BMDMs treated with caspase-1 inhibitor showed a severe reduction in IL-1β secretion (Figures 4B,D, respectively). Moreover, as caspase-8 has been associated with the non-canonical processing of IL-1β in response to *C. albicans, A. fumigatus*, and *C. neoformans* infection, we further tested caspase-8 inhibitor Z-IETD-FMK with WT BMDMs infected with *F. pedrosoi* hyphae (34, 35). Interestingly, cells treated with caspase-8 inhibitor decreased IL-1β production, although not as dramatically as cells undergoing caspase-1 inhibition (Figure 4D). TNF-α secretion was not affected in the inhibition/absence of the aforementioned caspases (Figures 4C,E). In addition to its role in maturing inflammasome-derived cytokines, caspase-1 mediate an inflammasome-dependent cell death mechanism termed pyroptosis, which leads to plasma membrane lysis and release of cytoplasmic content, including lactate dehydrogenase (LDH). Interestingly, we observed no extracellular release of LDH from BMDMs infected with *F. pedrosoi* hyphae nor conidia, which suggests that pyroptosis does not occur in our model (data not shown). Thus, our data demonstrate that IL-1β secretion by BMDMs in response to *F. pedrosoi* hyphae infection depends on caspase-1 and, to a lesser extent, on caspase-8 activity.

### *F. pedrosoi* Hyphae Activate the NLRP3 Inflammasome, Which Depends on K^+^ Efflux, ROS Production, Phagolysosomal Acidification/Disruption, and Cathepsin B Release

**Figure 5:**
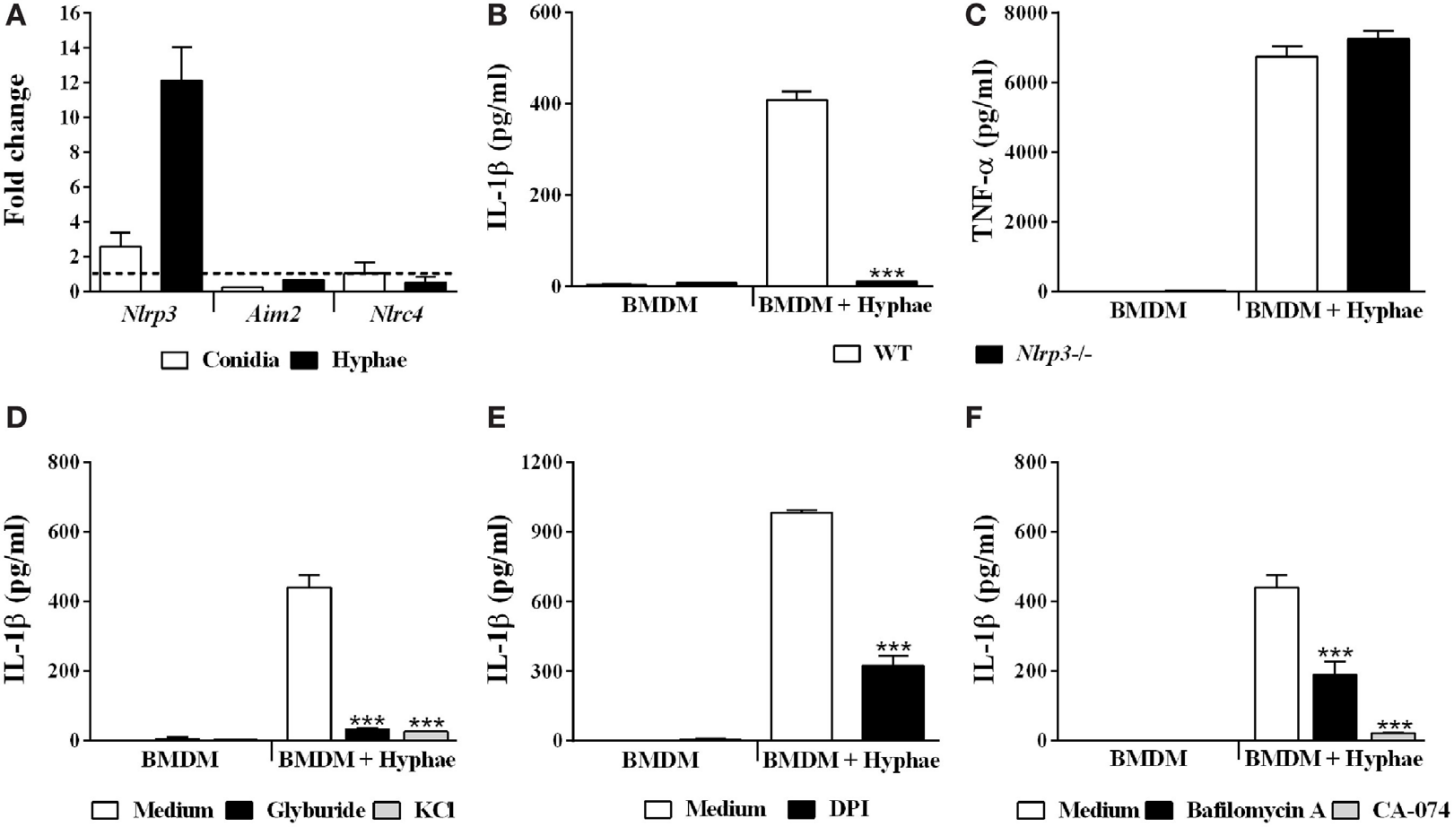
*Fonsecaea pedrosoi* hyphae activate the NLRP3 inflammasome, which depends on K^+^ efflux, ROS production, phagolysosomal acidification, and cathepsin B release. Bone marrow-derived macrophages (BMDMs) were infected with conidia or hyphae for 6 h and, thereafter, were lysed for RT-qPCR analysis to quantify gene expression of indicated genes **(A)**. BMDMs obtained from wild-type (WT) or *NLRP3^−/−^* mice were infected with *F. pedrosoi* hyphae for 24 h **(B,C)**. WT BMDMs were pretreated for 2 h with glyburide (150 µM) or KCl (50 mM) **(D)**, DPI (20 µM) **(E)**, and bafilomycin A (250 nM) or CA-074 (50 µM) **(F)**. Then, cells were infected with *F. pedrosoi* hyphae for 24 h. The supernatant of 24 h cell culture assays **(B–F)** was evaluated for IL-1β by ELISA. Data shown are mean ± SEM and are representative of two to three independent experiments. ***p < 0.001, compared to WT-infected untreated cells.

With respect to inflammasome activation upon fungal infections, only the inflammasome receptors AIM2, NLRC4 and, mainly, NLRP3, are related to engaging and triggering IL-1β caspase-dependent cleavage (36). Thus, we first evaluated the gene transcription of these receptors in BMDMs upon hyphal infection, and only *Nlrp3* was upregulated (Figure 5A). To evaluate whether NLRP3 participates in IL-1β secretion in BMDMs stimulated with hyphae, we used BMDMs obtained from NLRP3-deficient mice. In NLRP3 absence, IL-1β secretion was almost completely abolished (Figure 5B), whereas inflammasome-independent TNF-α production remained unchanged (Figure 5C).

NLRP3 is activated in response to a myriad of microbial infections through the sensing of their common-induced cellular disturbances, principally potassium efflux, ROS production, phagolysosomal acidification, and cathepsin B release (37). In order to assess whether the aforementioned mechanisms of NLRP3-inflammasome activation are required for our model, we impaired cell potassium efflux by cell treatment with glyburide, an ATP-sensitive K^+^ channel inhibitor, and also by addition of exogenous KCl. Under both conditions, we observed a drastic decrease in IL-1β levels (Figure 5D). To verify the role of ROS production, we used DPI, a blocker of ROS derived from the phagosome and mitochondria. In response, IL-1β secretion was impaired (Figure 5E). However, reduction in TNF-α secretion was also observed (data not shown). Furthermore, we measured IL-1β levels in cultures of BMDMs treated with bafilomycin A and CA-074Me, inhibitors of phagolysosomal acidification and cathepsin B cytosolic activity, respectively, which succeeds phagolysosomal damage and subsequent disruption. This inhibitory assay indicated that IL-1β secretion is strongly dependent on cathepsin B release and influenced by phagolysosomal acidification (Figure 5F). Furthermore, since cathepsin B release requires phagolysosome membrane permeabilization, we incubated BMDMs with FITC-dextran and evaluated whether it leaks into the cytosol (Figure S2 in Supplementary Material). Internalized FITC-dextran diffused into the cytosol after *F. pedrosoi* challenge, as previously described for *C. neoformans* infection (35), which suggests that disruption of the phagolysosome caused by *F. pedrosoi* preceded cathepsin B release in order to induce IL-1β secretion. Altogether, our data show that *F. pedrosoi* hyphae activate the NLRP3 inflammasome in BMDMs, which depends on K^+^ efflux, ROS production, phagolysosomal acidification, and disruption followed by cathepsin B release. All cell treatments used above were previously successfully tested (27) to exclude the reduction of IL-1β due to cytotoxic effects.

### Inflammasome Activation Does Not Promote *In Vitro* Fungicidal Activity of BMDMs Challenged with *F. pedrosoi* Conidia or Hyphae

**Figure 6:**
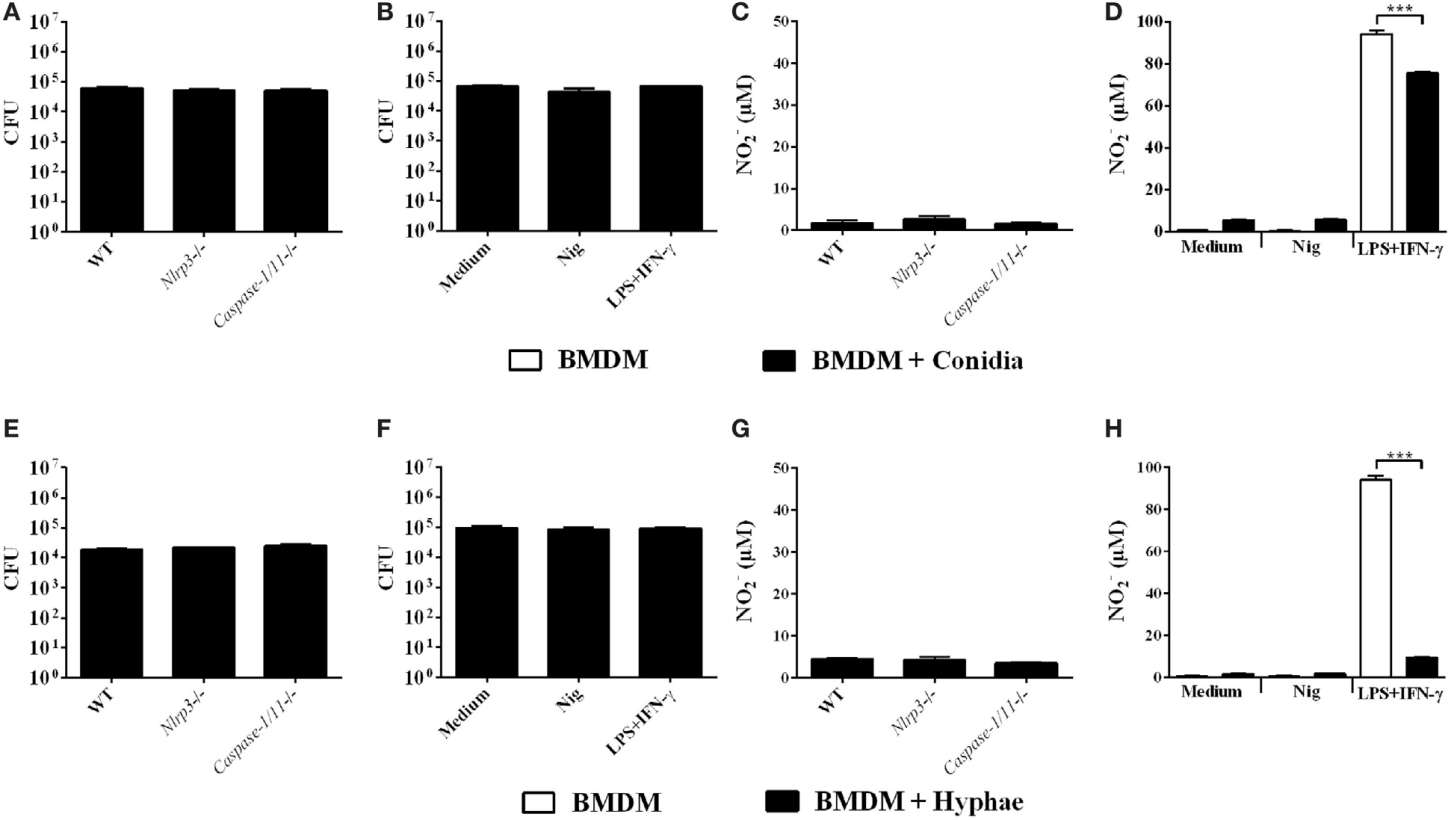
Inflammasome activation does not promote *in vitro* fungicidal activity of bone marrow-derived macrophages (BMDMs) challenged with *Fonsecaea pedrosoi* conidia or hyphae. BMDMs were infected for 24 h with *F. pedrosoi* conidia **(A–D)** or hyphae **(E–H)**. Then, culture supernatant was collected for nitrite measurement by Griess reagent, whereas infected cells were lysed to perform colony-forming unit (CFU) analysis. CFU **(A,E)** and ${\text{NO}}_{2}^{-}$ **(C,G)** values of an assay with infected wild-type (WT), *NLRP3^−/−^* and *Caspase-1^−/−^* BMDMs. CFU **(B,F)** and ${\text{NO}}_{2}^{-}$ **(D,H)** values of an assay with non-infected (white bars) and infected (black bars) WT BMDMs co-stimulated (or not) with both LPS and IFN-γ (500 and 20 ng/mL added 3 h before fungal infection, respectively) or nigericin (20 µM during the last 60 min of incubation). Data shown are mean ± SEM and are representative of three independent experiments. ***p < 0.001, between groups indicated by brackets.

Some studies have demonstrated that inflammasome activation drives host protective immune responses against fungal pathogens, such as *C. albicans, C. neoformans*, and *P. brasiliensis* (36). Thus, after clarifying the mechanisms of inflammasome activation in BMDMs infected with *F. pedrosoi*, we aimed to verify whether this is reflected in enhanced fungicidal activity by BMDMs against *F. pedrosoi* conidia and hyphae. To this end, we infected WT, *Caspase-1/11^−^*^/^*^−^* and *Nlrp3^−^*^/^*^−^* BMDMs for 24 h and thereafter performed the CFU assay. We observed no differences in fungal burden among experimental groups (Figures 6A,E), which we already expected for infection with conidia, since this morphotype alone is unable to activate the inflammasome in BMDMs, as demonstrated in the present study (Figure 2A). We did not observe NO production in these groups (Figures 6C,G). Therefore, we went further and co-stimulated infected BMDMs with nigericin to promote strong inflammasome activation, as shown in Figure 2A, and then performed the CFU assay. Nevertheless, the inducible burst in inflammasome activity was not accompanied by diminished recovery of viable fungus from BMDMs infected with conidia or hyphae (Figures 6B,F). Interestingly, a control group treated simultaneously with LPS and IFN-γ showed no difference in fungal killing (Figures 6B,F), despite the induced NO production (Figures 6D,H). This can be explained in part because *F. pedrosoi* morphotypes, especially hyphae, inhibited NO production (Figures 6D,H).

### NLRP3 Inflammasome Does Not Contribute to Fungal Clearance in a Murine CBM Model

**Figure 7:**
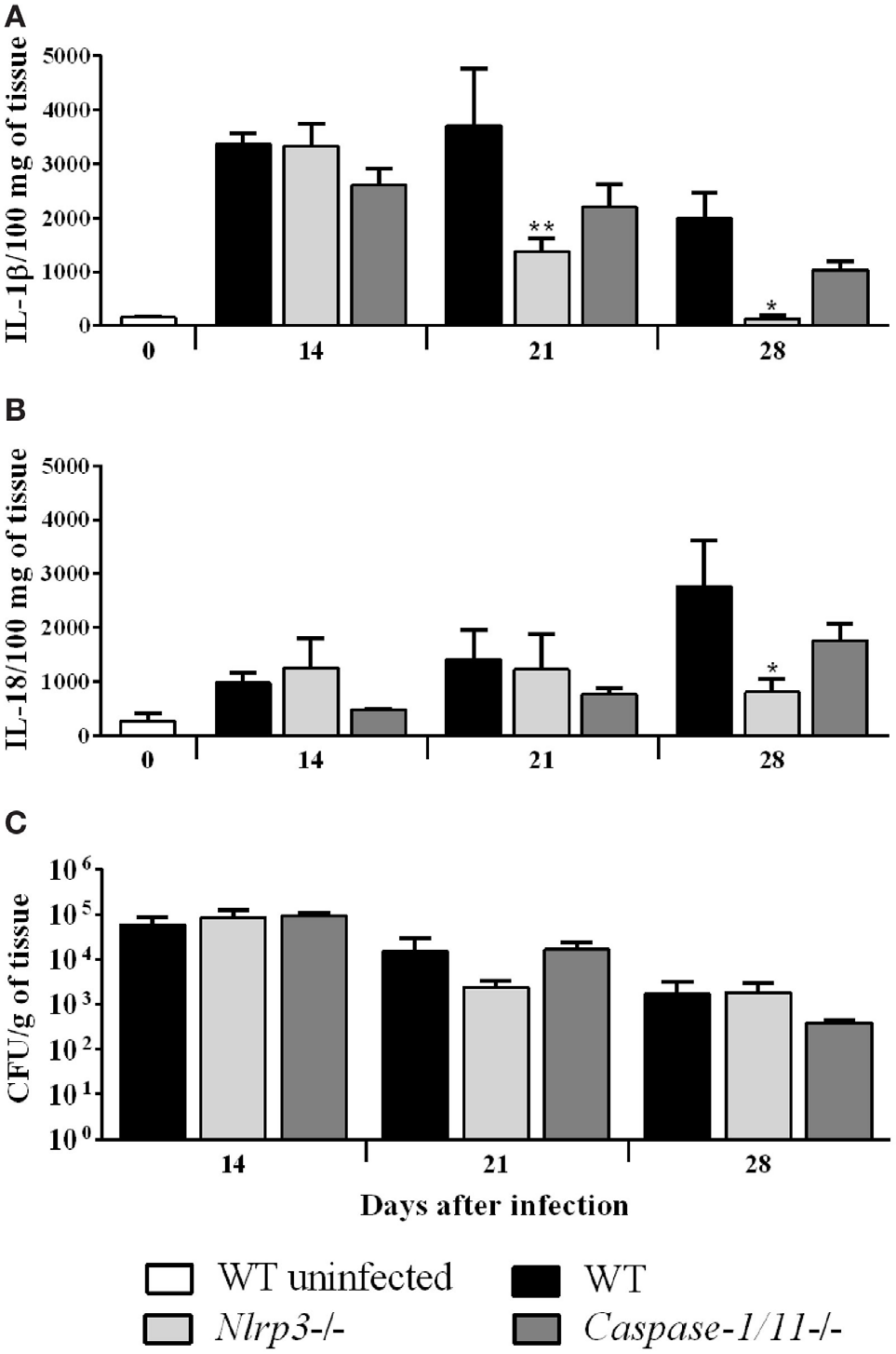
NLRP3 inflammasome does not contribute to fungal clearance in a murine chromoblastomycosis model. Wild-type (WT), *Nlrp3^−/−^* and *Caspase-1/11^−/−^* C57BL/6 mice (*n* = 4) were infected in both hind footpads with 1 × 10^6^ *Fonsecaea pedrosoi* propagules. WT uninfected mice were used as control. Mice were euthanized at the indicated days postinfection, and the footpad tissue homogenate was assessed for both cleaved and uncleaved IL-1β **(A)** and interleukin-18 **(B)** by ELISA, and plated for colony-forming unit analysis **(C)**. Data shown are mean ± SEM and are representative of two independent experiments. *p < 0.05; **p < 0.01, compared to WT infected.

In order to verify whether *F. pedrosoi* could activate the inflammasome *in vivo*, and to further investigate the functional role of the inflammasome in an experimental model of CBM, we infected WT, *Nlrp3* and *Caspase-1/11-*deficient mice subcutaneously in the footpad with a mixed inoculum of fungal propagules consisting of conidia and hyphae. WT-infected mice showed a sustained production of IL-1β at all the time points analyzed, which was reduced at 21 and 28 days postinfection (d.p.i.) in *Nlrp3^−^*^/^*^−^* mice (Figure 7A). In addition, we observed significant levels of IL-18 only in an advanced stage of remission of the disease, at 28 d.p.i., with defective cytokine production in *Nlrp3^−^*^/^*^−^* mice (Figure 7B). There was no difference in the production of both cytokines between WT and *Caspase-1/11^−^*^/^*^−^* mice (Figures 7A,B). Interestingly, the production of IL-1β and IL-18, regulated by NLRP3 and Caspase-1/11 in response to *F. pedrosoi* hyphae (Figures 4 and 5), did not affect the fungal load of the footpad, as we did not observe statistical differences in CFU assays among WT and both groups of knockout mice, at any of the times analyzed (Figure 7C). Histological examination of infected tissues from all groups show similar features as indicated by the presence of exudative areas and inflammatory infiltrates early as 14 d.p.i., which diminished over time and was accompanied by tissue remodeling and repair (Figure S3A in Supplementary Material). Consistently with CFU and histological analysis, there was no difference in morphometric measurements of the injured footpad among experimental groups (Figure S3B in Supplementary Material).

These data indicate that *F. pedrosoi* induces the production of IL-1β and IL-18 in the course of a subcutaneous experimental infection model, but this production does not affect the fungal clearance.

## Discussion

Studies examining innate immune cell recognition of *F. pedrosoi* are scarce. Although the involvement of CLRs and TLRs in this process is relatively well known, cytoplasmic NLRs had never been considered. In this study, we demonstrate that the main agent of CBM, *F. pedrosoi*, activates NLRP3 inflammasome-dependent secretion of IL-1β and IL-18 in phagocytes and *in vivo*. Moreover, the mechanisms underlying this process encompass Syk-coupled CLR receptors, caspase-1 and caspase 8 proteolytic activity and several DAMPs.

One key aspect shown to determine NLRP3 inflammasome activation during fungal infection is morphogenesis. We show here that live *F. pedrosoi* hyphae, and not conidia, were able to induce the production of mature IL-1β and IL-18 in BMDMs, despite the fact that both cell types prompted the upregulation of IL-1β transcripts (pro-IL-1β). In addition, only hyphal cells induced IL-1β secretion in human monocytic THP-1 cells. Similarly, activation of NLRP3 inflammasome and cytokine secretion in macrophages challenged with *A. fumigatus* was achieved only with hyphae. This suggests that an invading aggressive hyphal form may be necessary to trigger the inflammatory response (23). Actually, in lung tissues of NLRP3-deficient mice, minimal inflammatory reaction to *Aspergillus* hyphae infection is observed (19). Further, *F. pedrosoi* swollen conidia, an intermediate stage of conidia-into-hyphae germination, were not able to induce mature IL-1β production, as also shown in *A. fumigatus*-infected macrophages (23). Interestingly, inflammasome-independent cytokines, such as TNF-α, are more induced by *Aspergillus* swollen conidia compared to inactive conidia, and cell wall PAMPs are transiently displayed and detected by macrophage PRRs (38, 39). In this context, future studies are warranted for the identification of cell wall composition/PAMPs exposure modification during *F. pedrosoi* germination. In *C. albicans*, although the switch from yeast to hyphal morphogenesis was initially believed to be indispensable for the activation of the NLRP3 inflammasome (17), recent studies have showed that the remodeling of its cell wall leading to PAMPs exposure during phagocytosis is the main factor associated with inflammasome activation (40, 41).

Another important aspect that influences NLRP3 inflammasome activation during infectious processes is the host cell type. Indeed, differently from what is shown in macrophages, *F. pedrosoi* conidia alone were able to induce mature IL-1β production in dendritic cells. In addition, when nigericin (bacterial pore formation toxin) was added to macrophage cell culture, a significant amount of IL-1β secretion was observed, corroborating the fact that conidia induce pro-IL-1β production but fail to provide inflammasome-activating second signal in macrophages. Consistent with this observation, our group previously showed that *P. brasiliensis* can trigger pro-IL-1β production in macrophages and dendritic cells, but it was only in the latter that infection resulted in IL-1β maturation and release (27). Also, activation of NLRP3 inflammasome and production of mature IL-1β in macrophages infected with *C. albicans* yeast occurred only after a priming step with LPS (17, 42) whereas the fungus alone induced mature cytokine release in the dendritic cell (16). These results are in line with the fact that NLRP3 concentration is critical to the efficacy of inflammasome activation (31), and NLRP3 protein levels under steady-state conditions and after PRR engagement are higher in dendritic cells when compared with macrophages (43, 44). Furthermore, *in vivo* murine splenic conventional dendritic cells reveal constant high *Nlrp3* transcript levels (45). Thus, both fungal form and phagocyte type are critical factors that dictate NLRP3 inflammasome activation in fungal infections.

Priming is the first step required for inflammasome activation, and this process is generally associated with PRRs engagement by PAMPs leading to pro-IL-1β, pro-IL-18, and NLRP3 production. CLRs are the major PRRs family for the recognition of fungal carbohydrate residues and have been associated with the priming step of inflammasome activation in several fungal infections (46). For instance, the dectin-1 receptor is necessary for the production of pro-IL-1β and IL-1 β in murine and human macrophages and dendritic cells infected with *Microsporum canis, C. albicans*, and *Malassezia* spp. (42, 47, 48). Also, signaling mediated by dectin-2 is required for the production of IL-1β in dendritic cells infected with both hyphae and conidia of *C. albicans* (49). Interestingly, dectin-1 and dectin-2 have been associated with the induction of cytokine secretion, including inflammasome-dependent IL-1β, in a collaborative manner. Dectin-1 and dectin-2 double-deficient dendritic cells infected with *Trichophyton rubrum* or *H. capsulatum* have impaired secretion of IL-1β when compared to WT cells and cells with either receptor deficiency alone (21, 50). Moreover, using blocking antibodies for dectin-1 or dectin-2 in single or double dectin-1/dectin-2 deficient cells, Chang et al. (21) elegantly demonstrated that dectin-2 was the major receptor for inflammasome activation in *H. capsulatum*-infected dendritic cells. Although we did not employ double deficient cells, our results using cells lacking dectin-1 or dectin-2 clearly show that these CLRs play a significant role in inducing the secretion of IL-1β by macrophages infected with *F. pedrosoi* hyphae. This is in line with the fact that dectin-1 and dectin-2 are required for the development of Th17 cells in mice subcutaneously infected with *F. pedrosoi*, since IL-1β associated with IL-23 favors a Th17 response (11, 51).

Besides dectin-1 and dectin-2, we also demonstrated a role for dectin-3 in the induction of IL-1β secretion by BMDMs infected with *F. pedrosoi* hyphae. It is noteworthy that dectin-3 can form heterodimers with dectin-2 for sensing and mediation of host protective anti-*C. albicans* defense (32). In addition, dectin-3 is necessary for the development of vaccine-induced Th17 cells that are associated with protection against the fungal pathogen *Blastomyces dermatitidis* (52). The role of dectin-3 in the recognition of *F. pedrosoi* conidia has been evaluated using lacZ activity measurement in reporter cells coexpressing dectin-2/dectin-3 or expressing dectin-2 or dectin-3 alone (11). Only a weak response was detected in dectin-3 expressing cells, and cells coexpressing dectin-2/dectin-3 did not show an enhanced dectin-2 induced reporter activity. Furthermore, IL-6 production was not significantly altered in dectin-3-deficient cells. In this context, our results suggest that, contrary to conidia, *F. pedrosoi* hyphae may expose dectin-3 ligands leading to recognition and pro-inflammatory cytokine production by macrophages.

Upon PAMP binding, all three aforementioned CLRs initiate intracellular signaling pathways, and the Syk-CARD9-NFkB pathway is the best characterized and the most common during fungal infections (36, 46). For instance, Syk-dependent NLRP3 priming occurs in *C. albicans, A. fumigatus*-, *C. neoformans*- and *P. brasiliensis*-infected cells (16, 18, 23, 27). Indeed, a significant reduction in IL-1β secretion was shown here in macrophages infected with *F. pedrosoi* and treated with chemical inhibitors of Syk kinase and NFkB activity. This result parallels those showing *Syk* and *Nfkb1* upregulated transcripts induced by *F. pedrosoi* hyphae. It was noted that *Myd88* transcripts were also significantly induced by hyphae in macrophages, but their inhibition did not affect the production of IL-1β. Conversely, the secretion of the inflammasome-independent cytokine TNF-α relied upon dectin-1 and both Syk- and Myd88-mediated signaling, which is in accordance with previous studies showing that TNF-α production in macrophages treated with β-glucans (i.e., dectin-1 ligand) requires that Syk-dependent signaling synergizes with the Myd88 pathway (53).

Followed by priming, the proteolytic cleavage of pro-IL-1β into its mature form is canonically performed by the protease caspase-1, and this process is largely dependent on the assembly of the NLRP3 inflammasome in fungal infections (36, 54). Using a fluorescence-based assay, we demonstrated that only *F. pedrosoi* hyphae were directly able to activate caspase-1 in macrophages. In addition, caspase-1/11-deficient macrophages and macrophages treated with caspase-1 peptide inhibitor produced a significant, but not total, impairment in mature IL-1β secretion, suggesting that caspase-1-independent processes may also be involved. In fact, non-canonical processing of IL-1β has been shown to operate in phagocytes infected with fungal pathogens. *C. albicans* triggers caspase-8-mediated cleavage of pro-IL-1β independently of a NLR-containing inflammasome. This occurs *via* assembly of the CARD9–Bcl-10–MALT1 scaffold mediated by Syk signaling, followed by the recruitment of caspase-8 into this scaffold for direct IL-1β processing (34). Caspase-8 can also function in a NLRP3-dependent inflammasome activation manner, as demonstrated in phagocytes infected with *A. fumigatus* and *C. neoformans* (19, 35). The use of a specific caspase-8 peptide inhibitor shows here that this protease has a role in the processing of IL-1β in *F. pedrosoi-*infected macrophages. Given the fact that NLRP3-deficient macrophages completely failed to produce IL-1β upon *F. pedrosoi* challenge, the function of caspase-8 in our model probably relies on the NLRP3 inflammasome assembly instead of a direct processing activity.

Along with NLRP3, NLRC4 and AIM2-containing-inflammasomes have been implicated in the antifungal response (36, 54). Specifically, *C. albicans* triggers NLCR4 inflammasome exclusively in mucosal cells, and deficiency in this NLR results in impaired neutrophil infiltration in the mucosa in a murine model of oral infection (55). Conversely, the NLRP3 inflammasome is strictly required in phagocytes infected with *C. albicans* (16). Regarding *A. fumigatus*, this fungus induces cooperative and synergistic activation of the NLRP3 and AIM2 inflammasomes in phagocytes and in an experimental murine model of intranasal infection (19). Similarly, NLRP3 and AIM2 inflammasomes are activated by plasmodium hemozoin and DNA (56). In this context, we evaluated the transcript levels of *Nlrp3, Nlrc4*, and *Aim2* genes in BMDMs infected with the infective forms of *F. pedrosoi*. Hyphae induced the upregulation of *Nlrp3* transcripts only. These data, coupled with the fact that NLRP3-deficient infected macrophages do not produce IL-1β, as mentioned above, indicate that multiple inflammasome activation is probably not in use in our fungal model. At the same time, these results also suggest that, unlike in *A. fumigatus*, there is a poor availability of *F. pedrosoi* dsDNA (the main AIM2 ligand) in the cytosol of infected cells. Nevertheless, the use of mice deficient in NLRC4 and AIM2 would shed light on this issue.

Although not fully characterized, cellular stresses that induce endogenous DAMPs have been associated with NLRP3 assembly and inflammatory caspase activation. Among them, K^+^ efflux due to stimulation of the ATP-sensitive K^+^ channel, ROS generation, and lysosome rupture are usually presented (15). Using chemical inhibitors, we demonstrated that *F. pedrosoi* hyphae promote these intracellular disturbances in macrophages, leading to inflammasome activation. Both K^+^ efflux and ROS are the main cellular disturbances associated with NLRP3 activation in phagocytes infected with diverse fungi, encompassing those causing dermathophytoses (47, 48, 57), and invasive mycosis (16, 18, 21, 27). Specifically, potassium efflux is considered a common unifying pathway for NLRP3 inflammasome complex activation triggered by numerous NLRP3 stimuli (58). As such, we tested two inhibitors of K^+^ efflux, glyburide and KCl. Both treatments resulted in a significant impairment of IL-1β secretion by macrophages, reinforcing the central role of intracellular K^+^ level reduction in the regulation of NLRP3 inflammasome activation. Similarly, the use of DPI, an inhibitor of NADPH oxidase-dependent and mitochondria-derived ROS production, also led to diminished IL-1β production. It is noteworthy that ROS generation is apparently not directly associated with the NLRP3 activation (i.e., inflammasome assembly) step. Instead, it may affect the priming step, as several inhibitors of ROS generation or scavengers of ROS have been shown to inhibit NFκB-mediated transcription of NLRP3 and pro-IL-1β (16, 59). Indeed, besides IL-1β, the production of the inflammasome-independent NFκB-dependent TNF-α was abrogated with the treatment with DPI in macrophages infected with *F. pedrosoi* (data not shown). In addition to ROS and K^+^ efflux, disruption of the phagolysosomal membrane, leading to impairment of phagolysosomal acidification and leakage of enzymes such as cathepsin B into the cytoplasm results in NLRP3 activation (15). Therefore, macrophages infected with *F. pedrosoi* were treated with bafilomycin, which inhibits the vacuolar H^+^ ATPase, or CA-074, which in turn inhibits cathepsin B activity. The treatments resulted in a significant reduction in IL-1β secretion, similar to the activation of the inflammasome by several bacteria and particulate matter (15, 36). Regarding fungi, cathepsin B is not required for IL-1β production in dendritic cells infected with *C. albicans* or *A. fumigatus* (16, 19). Conversely, the production of this cytokine in macrophages infected with *C. albicans* is dependent on cathepsin B (17). In phagocytes infected with *P. brasiliensis*, dendritic cells, but not macrophages, require phagolysosomal disruption for NLRP3 inflammasome activation (27, 60). Thus, the requirement for phagolysosomal disruption in the assembly of NLRP3 inflammasome is not universal, as it is dependent on the cell type and fungal pathogen.

Macrophages are essential innate immunity cells that are critical for direct antifungal response. Macrophages, along with neutrophils and lymphocytes, are regularly observed within the chronic granulomatous lesions of CBM patients, displaying different degrees of maturation and activation, and they also form multinucleated giant cells that harbor fungi (29, 30). In addition, *F. pedrosoi* cells can be detected in intracytoplasmic vacuoles of skin macrophages (61). Thus, we investigated whether NLRP3 inflammasome activation would play a part in the microbicidal capacity of macrophages infected with *F. pedrosoi*. In BMDMs lacking NLRP3 or Caspase-1 and infected with hyphae or conidia, no significant difference in the fungal burden was observed. Since conidia alone are not able to induce inflammasome activation, we treated macrophages with nigericin for the induction of the second signal for inflammasome activation and consequently production of mature IL-1β. Macrophages infected with hyphae were also treated with nigericin, leading to enhanced IL-1β secretion. Again, fungal viability was not affected, reinforcing the supposition that the NLRP3 inflammasome has no role in modulating the ability of macrophages to kill *F. predosoi*. In fact, the killing process of *F. pedrosoi* by macrophages is rarely efficient, and it is neutrophils that are considered the main phagocyte cell with microbicidal activity against this fungus (62, 63). Several studies have demonstrated that *F. pedrosoi* is able to survive and proliferate in murine macrophages, and activated macrophages are only fungistatic (62, 64–66). One important factor associated with the inability of macrophages to kill *F. pedrosoi* is the inhibition of NO production by macrophages (66). In line with this finding, no significant levels of nitrite were detected in the supernatant of macrophage cultures infected with *F. pedrosoi* cells. Furthermore, macrophages infected with either conidia or hyphae and treated with IFN-γ and LPS were not fungicidal and their nitrite production was significantly impaired. Lack of properly activated macrophages may serve *F. pedrosoi* intracellular parasitism, leading to disease establishment and progression in susceptible hosts.

Since *F. pedrosoi* activates the NLRP3 inflammasome *in vitro*, we investigated whether *in vivo* activation occurs and the possible role of this process in controlling fungal infection. Employing a mouse model of subcutaneous *F. pedrosoi* infection (24, 67), WT-infected mice showed a sustained production of IL-1β at all the time points analyzed, which was significantly reduced in mice lacking NLRP3. In addition, the NLRP3 inflammasome was also required for the production of IL-18, as assessed in the footpad macerate at 28 d.p.i. These results suggest that NLRP3 sensing is required for the maturation of IL-1β and IL-18 in mice infected with *F. pedrosoi*. Surprisingly, the production of these cytokines was not significantly different in the footpad of caspase-1/11-deficient mice, although there was a tendency for IL-1β and IL-18 to be lower than in control mice. In macrophages infected with *F. pedrosoi*, caspase-1, and caspase-8 were required for the production of IL-1β. It is possible that *in vivo*, caspase-8 compensates for the lack of caspase-1. For instance, *C. neoformans* activates NLRP3-caspase-8 inflammasome in the absence of caspase-1 (35). Also, caspase-independent cleavage of pro-IL-1β may ensue, especially *via* neutrophil-derived serine proteases (68). This is in line with the fact that the chronic inflammatory response observed in murine and human CBM is characterized by a mononuclear granuloma modified by the influx of neutrophils, giving rise to a suppurative neutrophilic infiltrate (5, 24). In this context, in disseminated candidiasis where neutrophils are the main component of the infiltrates in inflammatory organs, IL-1β processing is probably mainly achieved by neutrophil-derived protease, rather than by caspase-1 (69, 70).

Differently from several models of systemic mycosis where NLRP3 inflammasome activation is associated with host protection and impairment of fungal growth (16–22), NLRP3 and caspase-1 deficient mice subcutaneously infected with *F. pedrosoi* developed to be self-healing in 30–40 days with a progressive fungal clearance. This finding is in accordance with the fact that infected mice lacking NLRP3 or Caspase-1 had similar histological and morphometric analyses of the footpad tissue when compared with control mice.

**Figure 8:**
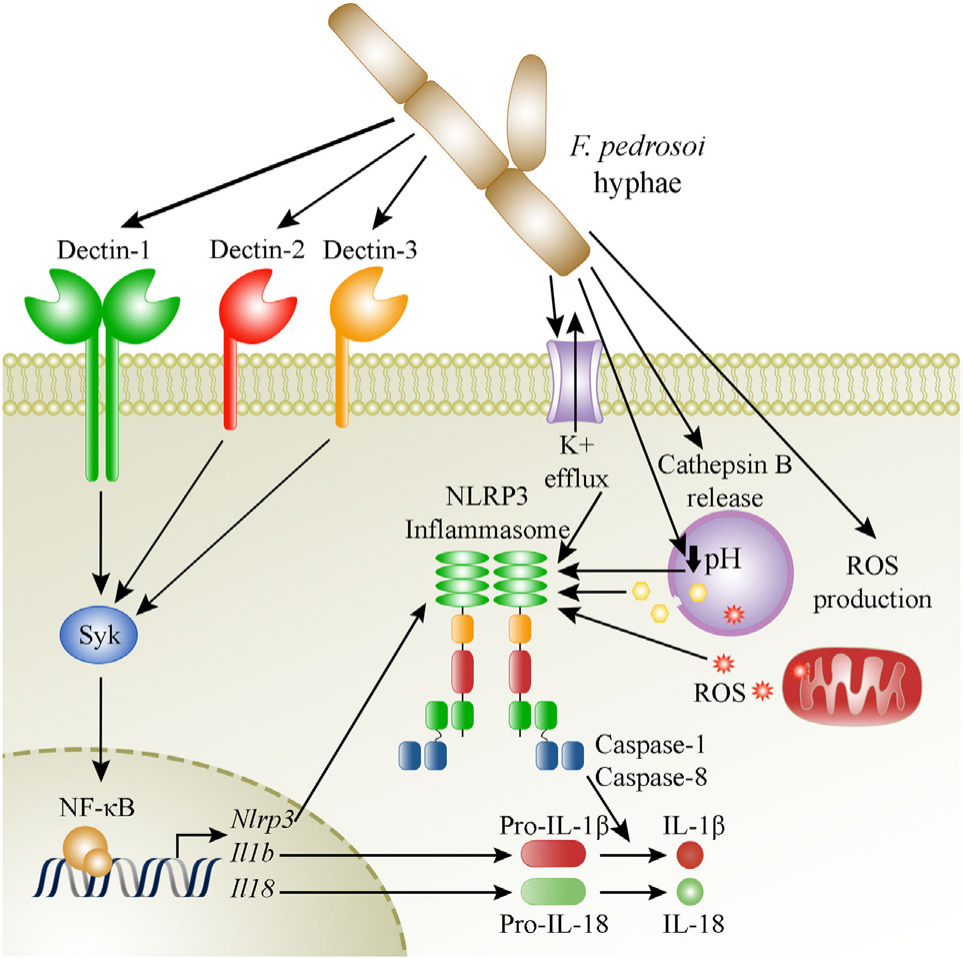
Suggested model of activation of the NLRP3 inflammasome by *Fonsecaea pedrosoi* hyphae in macrophages. *F. pedrosoi* hyphae induce macrophage priming by a canonical Syk-NF-kB signaling pathway coupled to dectin-1, dectin-2, and dectin-3 recognition of the fungus. This leads to NLRP3, pro-IL-1β, and pro-IL-18 production. Then, the NLRP3-inflammasome is activated as a result of potassium efflux, phagolysosome acidification, cathepsin B release, and ROS production, which subsequently leads to IL-1β and IL-18 processing and secretion by caspase-1 and caspase-8 dependent cleavage.

In conclusion, we revealed here the mechanisms of NLRP3 inflammasome activation in macrophages and in mice infected with the most important CBM etiologic agent *F. pedrosoi*, as depicted in our proposed model in Figure 8. Future studies employing other causative agents of CBM will contribute to a better understanding on the role of inflammasome activation in the pathogenesis of this neglected disease.

## Ethics Statement

All experimental procedures were approved by the Animal Ethics Committee of the University of Brasilia (UnBDoc number 134976/2014), and conducted according to the Brazilian Council for the Control of Animal Experimentation (CONCEA) guidelines.

## Author Contributions

Conceived and designed the experiments: RJAC, IMS, PHB, AHT, and ALB. Performed the experiments: RJAC, IMS, MSJ, AMMB, PHHVJ, SAMO, and LCL. Analyzed the data: RJAC, IMS, AHT, and ALB. Contributed to reagents/material/analysis tools: AHT and ALB. Contributed to Caspase-1 and NLRP3 KO mice: KGM. Wrote the paper: RJAC, AHT, and ALB.

## Conflict of Interest Statement

The authors declare that the research was conducted in the absence of any commercial or financial relationships that could be construed as a potential conflict of interest.

## Supplementary Material

Figure S1 *Fonsecaea pedrosoi* conidia reaches the swelling stage of germination. Flow cytometry analysis of *F. pedrosoi* conidia incubed for 6 h with phosphate-buffered saline, RPMI supplemented with 20% of SBF (R20 medium) or bone marrow-derived macrophages (BMDMs) for 6 and 24 h. Infected BMDMs were washed to discard nonphagocyted fungus and lysed to release intracellular fungi. After, conidia were fixed and evaluated by flow cytometry analysis. Commercial 2 and 3 µm sized-cytometry-beads were used as control. Dot plot side scatter (FSC) vs forward scatter (FSC) analysis of conidia **(A)**. Histogram analyses of conidia size **(B)**. Average conidia size using the median FSC and expressed as arbitrary units (au) **(C)**. Data shown are mean ± SEM and are representative of three independent experiments. ****p* < 0.001, between groups indicated by brackets; n.s., not significantly.

Figure S2 *Fonsecaea pedrosoi* hyphae infection cause phagolysosome membrane permeabilization contributing to inflammasome activation. Bone marrow-derived macrophages were seeded on glass bottom culture dishes with RPMI for 1 h for cell adhesion. After wash of unbound cells with warm RPMI, cells were incubated with 70 kDa FITC-dextran (Sigma-Aldrich; 2 mg/mL) in RPMI with 10% FBS for 2 h. The cells were washed and infected or not (medium) with *F. pedrosoi* hyphae (MOI 1) or *Cryptococcus neoformans* yeasts (MOI 2) opsonized with anti-GXM Ab. After 4 h of infection, non-phagocytosed fungi were washed and infection proceeded up to 20 h. After infection, cells were washed again, incubated with RPMI medium without phenol red supplemented with 10% FBS and stained with DAPI. Cells were washed, incubated with medium, and visualized by fluorescence microscopy. White arrowhead indicates internalized fungi.

Figure S3 NLRP3 or caspase-1/11 absence do not affect tissue response to *Fonsecaea pedrosoi* infection. Wild-type, *Nlrp3^−/−^* and *Caspase-1/11^−^*^/^*^−^* mice were injected subcutaneously with 1 × 10^6^ *F. pedrosoi* propagules into the hind footpad. For histology evaluation, infected mice were euthanized at the indicated days after infection and fragments of tissues were fixed with 10% phosphate-buffered formalin and embedded in paraffin. Paraffin-sectioned samples were stained with Hematoxilin and Eosin (H&E) and analyzed under a Zeiss inverted microscope **(A)**. During infection, the injured tissue was measured every 3 days with a caliper for morphometric examination **(B)**. Data shown are mean ± SEM (*n* = 4) and are representative of two independent experiments.
