# Supplementary material for: The Major Chromoblastomycosis Etiologic Agent Fonsecaea pedrosoi Activates the NLRP3 Inflammasome
Source: Front Immunol. 2017 Nov 20;8:1572. doi: 10.3389/fimmu.2017.01572 (PMC5702042; doi:10.3389/fimmu.2017.01572)
Supplement: Supplementary file 1 [file Table_1.DOCX]

Table S1. Primers used for qRT-PCR.

| Gene | Forward primer (5’ to 3’) | Reverse primer (5’ to 3’) |
| --- | --- | --- |
| *Rps9* | CGCCAGAAGCTGGGTTTGT | CGAGACGCGACTTCTCGAA |
| *Il1b* | GTGTGTGACGTTCCCATTAGACA | CAGCACGAGGCTTTTTTGTTG |
| *Il18* | ACTGTACAACCGCAGTAATACG | AGTGAACATTACAGATTTATCCC |
| *Tnfa* | GTACCTTGTCTACTCCCAGGTTCTCT | GTGGGTGAGGAGCACGTAGTC |
| *Syk* | CTACCTGCTACGCCAGAGC | GCCATTAAGTTCCCTCTCGATG |
| *Myd88* | actggcctgagcaactagga | cgtgccactacctgtagcaa |
| *Nfkb1* | AGCCAGCTTCCGTGTTTGTT | AGGGTTTCGGTTCACTAGTTTCC |
| *Dectin1* | TAATCTCTGCCCCCAAAACC | AACTGCTTCGACCCAGACCT |
| *Dectin2* | ACCCCTGACCTTCTGAACATACAC | TGAGCCCCCATCTGAACACA |
| *Dectin3* | TTGTGACCCAGCTTTTGGAT | TCCAGACCCATTTTTCATGG |
| *Nlrp3* | ATTACCCGCCCGAGAAAGG | TCGCAGCAAAGATCCACACAG |
| *Aim2* | TGGCAAAACGTCTTCAGGAGG | GATGCAGCAGGACTCATTTCA |
| *Nlrc4* | ATCGTCATCACCGTGTGGAG | GCCAGACTCGCCTTCAATCA |
