# Supplementary figures and images for: The Major Chromoblastomycosis Etiologic Agent Fonsecaea pedrosoi Activates the NLRP3 Inflammasome
Source: Front Immunol. 2017 Nov 20;8:1572. doi: 10.3389/fimmu.2017.01572 (PMC5702042; doi:10.3389/fimmu.2017.01572)

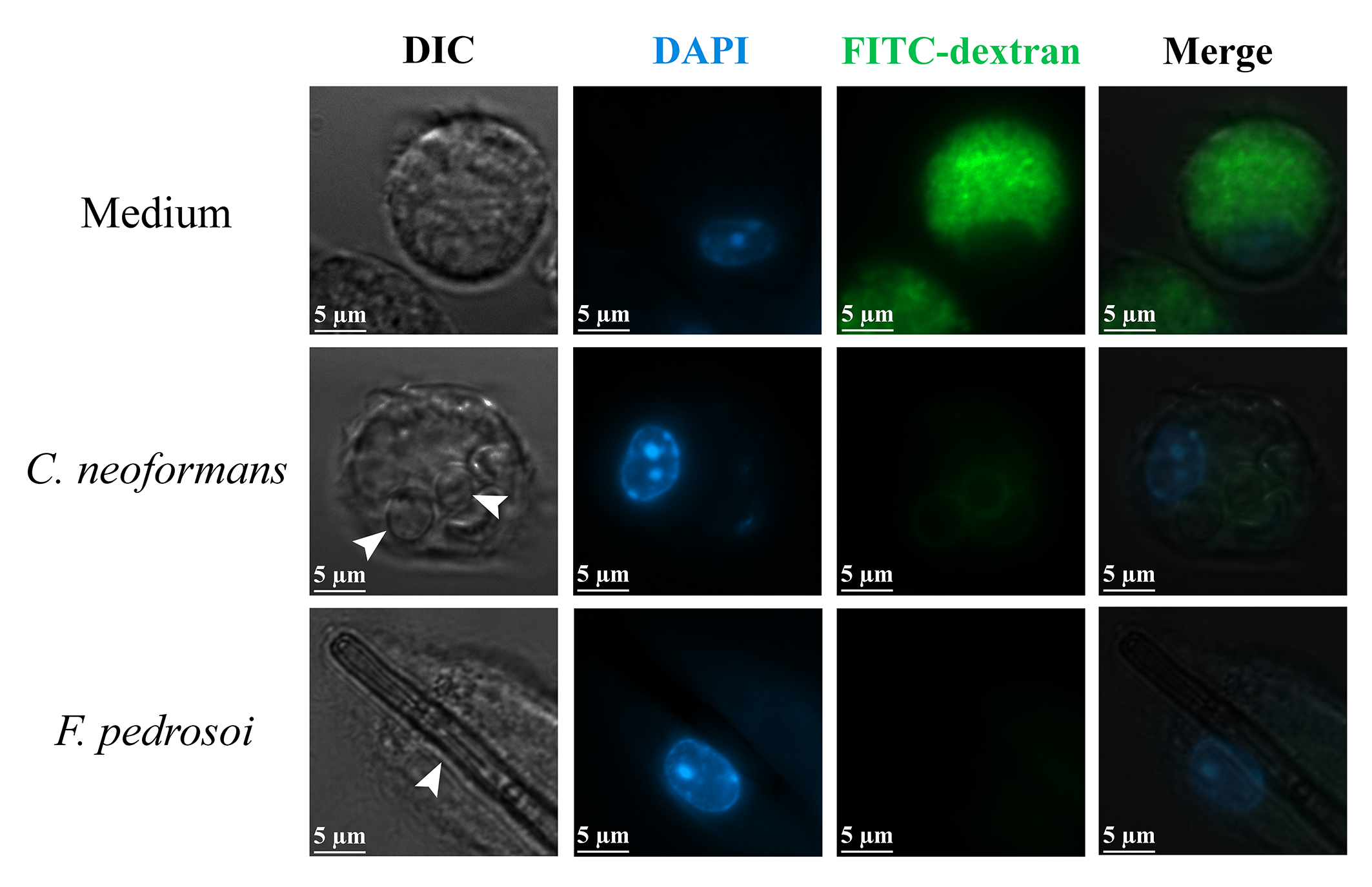

Supplement: Figure S2 — Fonsecaea pedrosoi hyphae infection cause phagolysosome membrane permeabilization contributing to inflammasome activation. Bone marrow-derived macrophages were seeded on glass bottom culture dishes with RPMI for 1 h for cell adhesion. After wash of unbound cells with warm RPMI, cells were incubated with 70 kDa FITC-dextran (Sigma-Aldrich; 2 mg/mL) in RPMI with 10% FBS for 2 h. The cells were washed and infected or not (medium) with F. pedrosoi hyphae (MOI 1) or Cryptococcus neoformans yeasts (MOI 2) opsonized with anti-GXM Ab. After 4 h of infection, non-phagocytosed fungi were washed and infection proceeded up to 20 h. After infection, cells were washed again, incubated with RPMI medium without phenol red supplemented with 10% FBS and stained with DAPI. Cells were washed, incubated with medium, and visualized by fluorescence microscopy. White arrowhead indicates internalized fungi. [file Image_2.TIF]

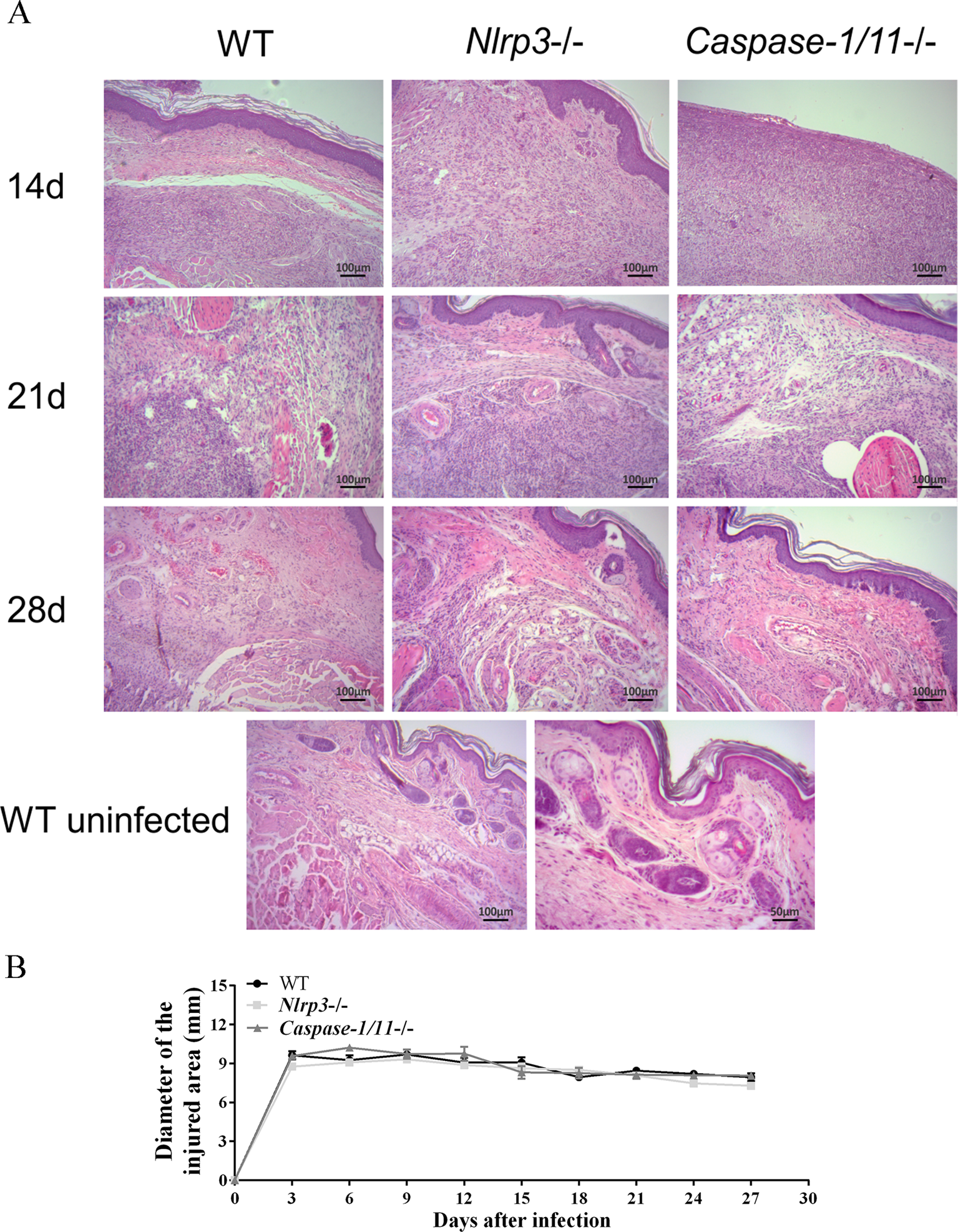

Supplement: Figure S3 — NLRP3 or caspase-1/11 absence do not affect tissue response to Fonsecaea pedrosoi infection. Wild-type, Nlrp3−/− and Caspase-1/11−/− mice were injected subcutaneously with 1 × 10^6^ F. pedrosoi propagules into the hind footpad. For histology evaluation, infected mice were euthanized at the indicated days after infection and fragments of tissues were fixed with 10% phosphate-buffered formalin and embedded in paraffin. Paraffin-sectioned samples were stained with Hematoxilin and Eosin (H&E) and analyzed under a Zeiss inverted microscope (A). During infection, the injured tissue was measured every 3 days with a caliper for morphometric examination (B). Data shown are mean ± SEM (n = 4) and are representative of two independent experiments. [file Image_3.TIF]
